# Diapause is associated with a change in the polarity of secretion of insulin-like peptides

**DOI:** 10.1038/ncomms10573

**Published:** 2016-02-03

**Authors:** Yohei Matsunaga, Yoko Honda, Shuji Honda, Takashi Iwasaki, Hiroshi Qadota, Guy M. Benian, Tsuyoshi Kawano

**Affiliations:** 1 Department of Bioresources Science, The United Graduate School of Agricultural Sciences, Tottori University, Tottori-shi, Tottori 680-8553, Japan; 2 Section for Genomics for Longevity and Health, Tokyo Metropolitan Institute of Gerontology, Itabashi-ku, Tokyo 173-0015, Japan; 3 Department of Pathology, Emory University School of Medicine, Atlanta, Georgia 30322, USA

## Abstract

The insulin/IGF-1 signalling (IIS) pathway plays an important role in the regulation of larval diapause, the long-lived growth arrest state called dauer arrest, in *Caenorhabditis elegans*. In this nematode, 40 insulin-like peptides (ILPs) have been identified as putative ligands of the IIS pathway; however, it remains unknown how ILPs modulate larval diapause. Here we show that the secretory polarity of INS-35 and INS-7, which suppress larval diapause, is changed in the intestinal epithelial cells at larval diapause. These ILPs are secreted from the intestine into the body cavity during larval stages. In contrast, they are secreted into the intestinal lumen and degraded during dauer arrest, only to be secreted into the body cavity again when the worms return to developmental growth. The process that determines the secretory polarity of INS-35 and INS-7, thus, has an important role in the modulation of larval diapause.

## Abstract (web summary)

Insulin-like peptides INS-7 and INS-35 suppress larval diapause in Caenorhabditis elegans via unknown mechanism. Here, Matsunaga *et al.* show that the secretory polarity of both peptides changes in diapause, when these peptides are secreted into the intestinal lumen instead of the body cavity like in other larval stages.

## Graphical abstract

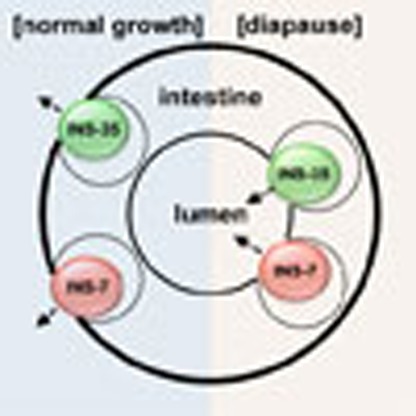

The insulin/IGF-1 signalling (IIS) pathway plays an important role in the regulation of larval diapause, the long-lived growth arrest state called dauer arrest, in *Caenorhabditis elegans* (*C. elegans*)^1^,^2^,^3^,^4^. DAF-2 is the sole insulin/IGF-1 receptor ortholog of the IIS pathway in *C. elegans*, and its mutation leads to dauer arrest^5^. Activation and inactivation of the DAF-2 receptor are modulated by insulin-like peptides (ILPs). To date, 40 ILPs have been identified as putative ligands and classified into type-α, type-β and type-γ according to the number and the position of disulphide bonds^6^. Type-β and type-γ ILPs play agonistic or antagonistic roles in the modulation of larval diapause: INS-6, -7 and DAF-28 (type-β) suppress dauer arrest^7^,^8^,^9^, whereas INS-1 (type-β), -17 and -18 (type-γ) promote dauer arrest^6^,^10^,^11^. However, it remained unclear whether type-α ILPs (total of 15 members) play agonistic or antagonistic roles in the modulation of larval diapause. We have found that the secretory polarity of INS-35 (type-α), which suppresses larval diapause, is changed in the intestinal epithelial cells at larval diapause. This ILP is secreted from the intestine into the body cavity during L1 and L2 larval stages. In contrast, it is secreted into the intestinal lumen and degraded during dauer arrest. Furthermore, it is secreted into the body cavity again when the worms return to developmental growth. Interestingly, the secretory polarity of INS-7 (type-β), which suppresses larval diapause, is also changed in the intestinal epithelial cells at larval diapause. These results suggest that larval diapause involves a change in the secretion polarity of INS-35 and INS-7 in the intestine. Here, we propose a model that the process, which determines the secretory polarity of ILPs, has an important role in the efficient modulation of larval diapause.

## Results and Discussion

### INS-35 critically suppresses dauer arrest in the intestine

**Figure 1:**
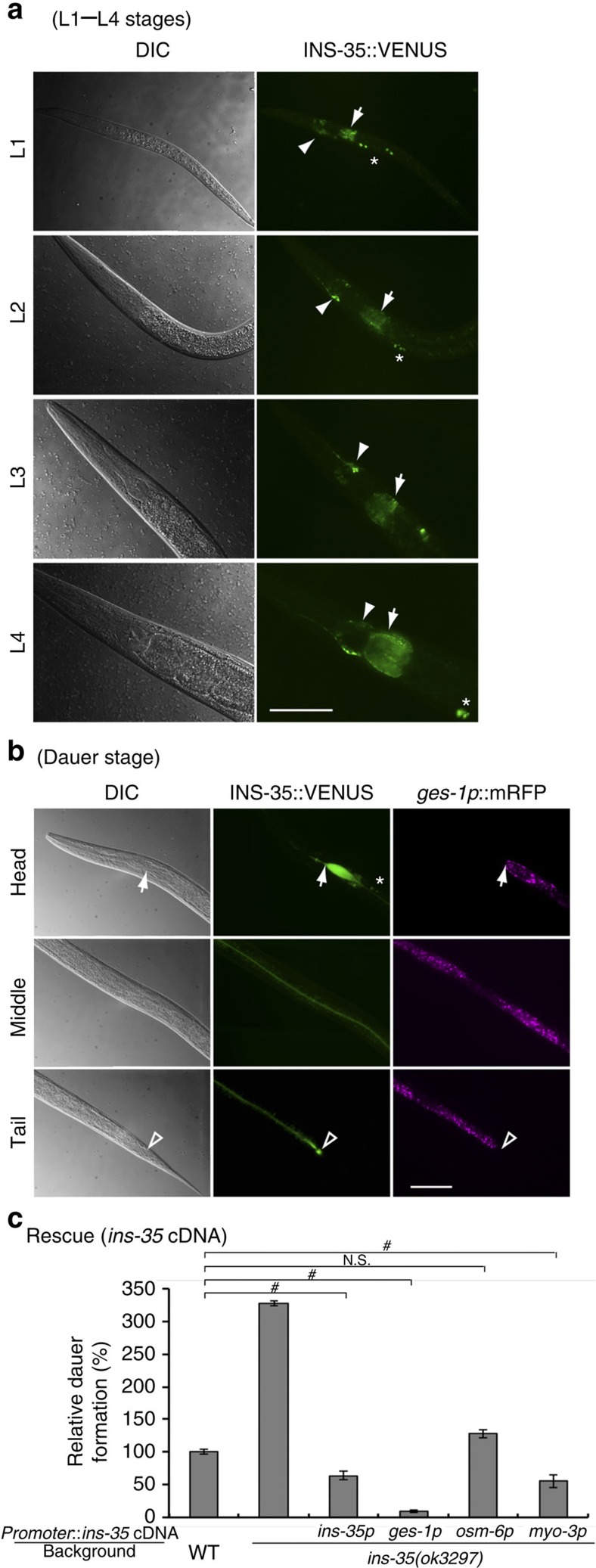
INS-35 markedly suppresses dauer arrest in the intestine. (**a**) Expression patterns of *ins-35p*::INS-35::VENUS from L1 to L4 stages. Solid arrowheads indicate amphid neurons. Asterisks indicate the coelomocytes. Arrows indicate the intestinal tissue. The scale bar, 50 μm. (**b**) Localization of *ins-35p*::INS-35::VENUS and *ges-1p*::mRFP at the day 1 dauer stage. Arrows indicate the intestinal valves. Hollow arrowheads indicate the anus. The asterisk indicates a pair of coelomocytes. The scale bar, 50 μm. (**c**) Relative percentages of dauer formation in each *ins-35* mutant in the presence of dauer-inducing pheromone compared with wild type are shown. Data are expressed as the mean±s.e.m. # *P*<0.05; NS, not significant. Error bars are defined as s.e.m. Multiple comparisons between groups were made using Dunnett's test. Detailed parameters including numbers, trials, *P* values, and values of mean±s.e.m. are shown in Supplementary Table 2.

To investigate whether type-α ILPs modulate larval diapause, we first performed RNAi-mediated knockdown^12^ of type-α insulin-like genes. We found that knockdown of only *ins-35* (ref. 13) resulted in statistically significant promotion of dauer arrest (Supplementary Table 1). This promotion was also shown by *ins-35*(*ok3297*) mutants in the presence of dauer-inducing pheromone^14^ (Fig. 1). In addition, it has been reported that *ins-35*(*ok3297*) mutants show promotion of dauer arrest under high-temperature (27 °C), *daf-2*(*e1368*) or *daf-28*(*sa191*) background^15^. *ins-35*(*ok3297*) has a 664 bp deletion including the second exon, indicating that *ins-35*(*ok3297*) is a loss of function mutant (Supplementary Fig. 1). Therefore, the normal function of INS-35 is to suppress larval diapause. Next, to examine the cellular expression pattern of *ins-35*, we generated a transgenic worm expressing INS-35 fused to VENUS (ref. 16), a variant of yellow fluorescent protein, driven by the *ins-35* promoter region (translational fusion: *ins-35p*::INS-35::VENUS). From L1 to L4 stages, INS-35::VENUS fluorescence was observed in the intestine, chemosensory neurons and coelomocytes, which are scavenger cells that constitutively endocytose material secreted into the body cavity (pseudocoelom)^17^,^18^ (Fig. 1a). At the dauer stage, the fluorescence was observed in the intestine and coelomocytes (Fig. 1b). To verify the *ins-35* expression pattern, we also generated a transgenic worm expressing VENUS driven by the *ins-35* promoter region (transcriptional fusion: *ins-35p*::VENUS). At the non-dauer (L2-L3) stages, VENUS fluorescence was observed in the intestine and chemosensory neurons. At the dauer stage, fluorescence was observed in the intestine. As expected, we could not detect fluorescence in coelomocytes at both non-dauer and dauer stages likely due to absence of a signal sequence in VENUS (Supplementary Fig. 2). INS-35::VENUS fluorescence observed in the coelomocytes is likely to be due to uptake into coelomocytes of secreted INS-35::VENUS. Taken together, *ins-35* is expressed in the intestine and chemosensory neurons at L1 to L4 stages, and in the intestine at the dauer stage.

To assess where INS-35 suppresses dauer arrest, we expressed *ins-35* cDNA fragments in a tissue-specific manner in *ins-35*(*ok3297*). In the presence of dauer-inducing pheromone, *ins-35* cDNA expressed in chemosensory neurons partially rescued the phenotype of *ins-35*(*ok3297*) mutants. In contrast, *ins-35* cDNA expressed in the intestine or muscle cells completely rescued the phenotype of *ins-35*(*ok3297*) mutants, the same as *ins-35* cDNA expressed by its native promoter. Among them, *ins-35* cDNA expressed in the intestine completely suppressed dauer arrest, even more than wild type (Fig. 1c). This tendency was also observed in the tissue-specific expression of the *ins-35* ORF with the gene's ∼380 bp intron (Supplementary Fig. 3). Therefore, these results suggest that INS-35 critically suppresses dauer arrest in the intestine. Interestingly, although *ins-35* is not normally expressed in muscle cells, *ins-35* expression in muscle resulted in the suppression of dauer arrest. It has been reported that a secretory signal sequence GFP expressed in muscle is secreted into the pseudocoelom and then accumulates in coelomocytes^19^. INS-35::VENUS expressed in muscle cells also accumulated in coelomocytes at the L2 stage (Supplementary Fig. 4). The DAF-2 receptor is expressed in head neurons and in the intestine^20^,^21^, which is the tissue adjacent to the pseudocoelom. It is possible that INS-35 secreted from muscle cells into the pseudocoelom might suppress larval diapause by binding to DAF-2 receptors.

### INS-35 is secreted into the intestinal lumen at dauer arrest

**Figure 2:**
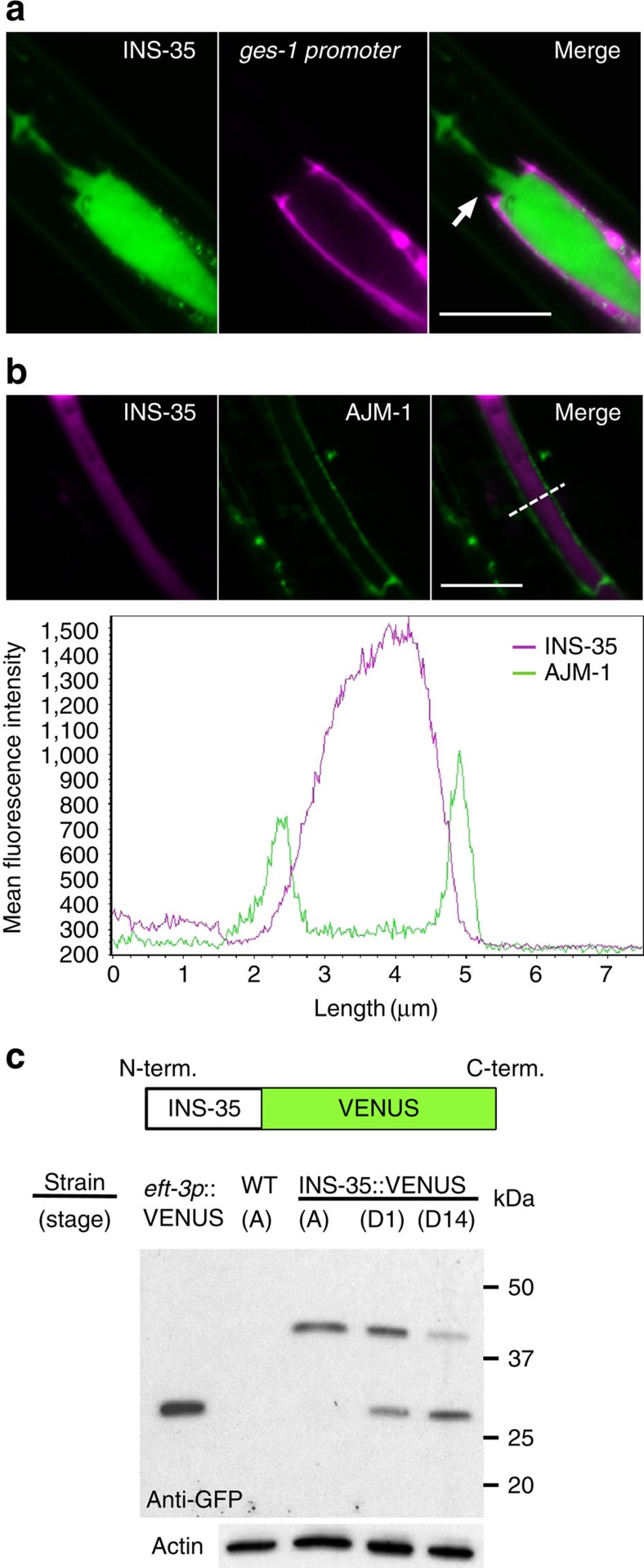
During dauer arrest, INS-35 is secreted into the intestinal lumen and is degraded. (**a**) Fluorescence images of INS-35::VENUS (left), *ges-1p*::mRFP (centre) and a merge (right) at the day 1 dauer stage. Arrows indicate the intestinal valve. Scale bar, 25 μm. (**b**) The top panels show images of INS-35::mRFP and AJM-1::GFP in the intestine at the day 1 dauer stage. Scale bar, 10 μm. The mean fluorescence intensity profile of AJM-1::GFP and INS-35::mRFP in the intestine is shown below. The dashed line indicates the cross section used to quantify fluorescence intensity indicated on the graph. (**c**) Western blot analysis using anti-GFP and anti-actin antibodies of extracts of *ins-35p*::*ins-35*::*venus* expressing worms: A, adult stage; D1, dauer stage at day 1; and D14, dauer stage at day 14. Images of the full blots reacted by anti-GFP or anti-actin antibodies are shown in Supplementary Fig. 6a,b.

To investigate how INS-35 modulates larval diapause, we observed the expression patterns of INS-35::VENUS at dauer arrest. Interestingly, INS-35::VENUS showed an accumulation in the intestinal area (Fig. 1b). To investigate where INS-35::VENUS accumulated, we first compared, in the same animal, the expression patterns of *ins-35* and *ges-1*, which encodes an esterase expressed in intestinal cells. mRFP expressed by the *ges-1* promoter (*ges-1p*::mRFP) was observed in intestinal cells. In contrast, most of the INS-35::VENUS was observed in the intestinal canal. This localization was especially broad in the anterior intestinal lumen (Fig. 2a). To obtain additional evidence that tagged INS-35 accumulates within the intestinal canal, we co-expressed INS-35::mRFP and AJM-1::GFP, as AJM-1 is a marker of the intestinal cell apical membrane^22^. As shown in Fig. 2b, intensity profiles of INS-35 and AJM-1 only partially overlap. Most of the INS-35::mRFP accumulated extracellularly between cells marked by AJM-1::GFP. These results indicate that INS-35 accumulated inside the intestinal canal after secretion from the apical membrane of intestinal cells at dauer arrest.

### INS-35 is degraded during dauer arrest

To elucidate why INS-35 accumulates in the intestinal canal, we first observed the pattern of the INS-35::VENUS signal as dauer larvae age. Fluorescence in neurons was not detectable from day 1 to 14 of dauer arrest. In contrast, fluorescence in the intestinal canal gradually decreased during dauer arrest (Supplementary Fig. 5), suggesting that INS-35::VENUS is degraded in the intestinal canal. To investigate possible degradation of INS-35, we next performed western blot analysis using *ins-35p*::*ins-35*::*venus* (Fig. 2c). In *ins-35p*::*ins-35*::*venus* expressing worms, an anti-GFP monoclonal antibody clearly detected INS-35::VENUS (∼42 kDa) in lanes corresponding to adults and individuals of dauer arrest. Significantly, a band of approximately 31 kDa, the size expected for VENUS, became visible in the lane corresponding to day 1 of dauer arrest, and at day 14, this band was even more prominent, whereas the INS-35::VENUS band was faint. To obtain additional evidence for the possible degradation in the intestine, we generated *ges-1p*::*ins-35* cDNA::*venus* expressing worms and an anti-INS-35 polyclonal antibody to perform western blot analysis (Supplementary Fig. 6c–e). The anti-GFP and the anti-INS-35 antibodies detected INS-35::VENUS in lanes corresponding to adults and individuals of dauer arrest. The anti-GFP antibody also detected a band of ∼31 kDa, the size expected for VENUS, in the lane corresponding to individuals at day 14 of dauer arrest (indicated as an asterisk). In contrast, the anti-INS-35 antibody did not detect either the band expected for VENUS or an 8 kDa band, the size expected for the INS-35 moiety (Supplementary Fig. 6c–e). These results suggest that the INS-35 moiety in INS-35::VENUS was degraded.

The 42 kDa band expected for INS-35::VENUS expressed from the *ges-1* promoter is brighter in day 14 dauers than in day 1 dauers (Supplementary Fig. 6c–e). In contrast, the 42 kDa band expressed from the *ins-35* promoter/*cis*-regulatory regions is weaker in day 14 dauers than in day 1 dauers (Fig. 2c). It is possible that this discrepancy is due to differences in promoter strength of *ins-35* and *ges-1* promoters. The *ins-35* promoter might be downregulated by the dauer program, whereas the *ges-1* promoter might be independent of the dauer program. To investigate this possibility, we compared the fluorescence intensity level of *ges-1p*::VENUS and *ins-35p*::VENUS during the non-dauer (L2-L3) and dauer stages. Fluorescence intensity level of *ges-1p*::VENUS does not change between the non-dauer and the dauer stages, whereas the level of *ins-35p*::VENUS is decreased in the dauer stage (Supplementary Fig. 7c,d). Consistent with our results, it has been reported that the level of *ins-35* mRNA is lower in the dauer stage than in the non-dauer (L2-L3) stages^23^. In addition, as shown in Supplementary Fig. 7a,b, *ins-35p*::VENUS was observed in a part of intestinal cells (most anterior side), whereas *ges-1p*::VENUS was observed in all intestinal cells. Therefore, the decrease in INS-35 is due to both dauer-dependent transcriptional and post-translational regulation, where the *ges-1* promoter interferes with both regulatory mechanisms. The dauer-independent *ges-1* promoter drives a much higher expression of INS-35::VENUS in all dauer intestinal cells, making this protein's degradation less evident (Supplementary Fig. 6c–e) when expressed from this promoter. Considering that the *ins-35* promoter only appears to drive gene expression in the anterior intestinal compartment (Supplementary Fig. 7b), it is possible that degradation of INS-35 is also more favored at the anterior part of the dauer intestinal lumen.

As an additional attempt to detect native INS-35, we performed western blot analysis using protein samples prepared from wild-type or *ins-35*(*ok3297*) animals, respectively. However, we could not detect any signal near 8 kDa (data not shown). It is possible that the amount of native INS-35 is quite low. Taken together, it is likely that INS-35 is secreted into the intestinal canal and degraded during dauer arrest.

### INS-7 is also secreted into the intestinal canal at dauer arrest

**Figure 3:**
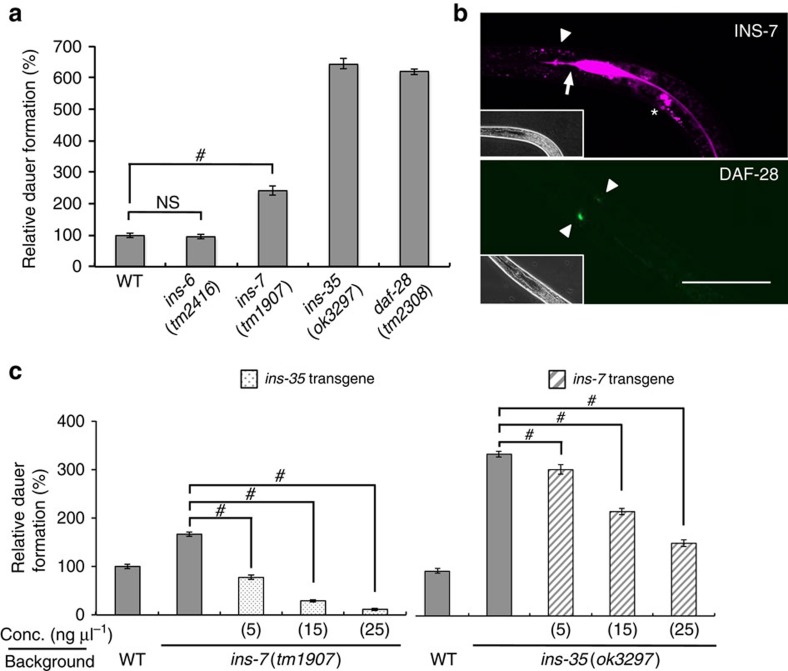
INS-7 is secreted into the intestinal lumen during dauer arrest, similar to INS-35. (**a**) Relative percentages of dauer formation in each knockout mutant in the presence of dauer-inducing pheromone compared with wild type are shown. (**b**) Fluorescence images of INS-7::mRFP (top) and DAF-28::VENUS (bottom) at the day 1 dauer stage. Solid arrowheads indicate amphid neurons. Arrow indicates the intestinal valve. Asterisk indicates coelomocytes. Scale bar, 50 μm. (**c**) Relative percentages of dauer formation in the mutants in the presence of dauer-inducing pheromone compared with wild type are shown (bypass experiments). *ins-7*(*tm1907*) and *ins-35*(*ok3297*) animals were injected with the indicated the concentrations of *ins-35* or *ins-7* transgenes (genome), respectively. Data are expressed as the mean±s.e.m. #, *P*<0.05; NS, not significant. Error bars are defined as s.e.m. Multiple comparisons between groups were made using Dunnett's test. Detailed parameters including numbers, trials, *P* values, and values of mean±s.e.m. are shown in Supplementary Table 2.

It is possible that other ILPs, which suppress larval diapause, are secreted into the intestinal canal and then degraded there during dauer arrest. To investigate this, we first verified the dauer mutant phenotypes of INS-6, -7 and DAF-28 (type-β) (refs 7, 8, 9) in the presence of dauer-inducing pheromone. We found that both *ins-7*(*tm1907*) and *daf-28*(*tm2308*) deletion mutants promoted dauer arrest under this condition, whereas *ins-6*(*tm2416*) deletion mutant did not (Fig. 3a). We subsequently generated transgenic worms expressing *ins-7p*::*ins-7*::*mrfp* or *daf-28p*::*daf-28*::*venus* and observed their expression at dauer arrest. DAF-28::VENUS was only expressed in chemosensory neurons. On the other hand, INS-7::mRFP was observed in the intestine, amphid neurons and coelomocytes, particularly in the intestinal canal, similar to INS-35::VENUS (Fig. 3b). In addition, the mean fluorescence intensity of INS-7::mRFP decreased in the intestinal canal from days 1 to 14 of dauer arrest (Supplementary Fig. 5). These results suggest that INS-7 seems to be degraded in the intestinal canal during dauer arrest, similar to INS-35. We also investigated whether the accumulation is only observed in dauer larvae. Transgenic animals expressing INS-35::VENUS and INS-7::mRFP were treated with a moderate concentration of pheromone, yielding ∼50% dauer formation. We checked the accumulation of the tagged ILPs in the intestinal lumens of 10 dauers and 10 non-dauers, respectively. Only the dauer larvae showed accumulation in the intestinal lumens (Supplementary Fig. 8a,b). INS-7 and INS-35 function to suppress dauer arrest and promote normal development. Perhaps, the accumulation and degradation of these ILPs in the lumenal space helps prevent normal development and maintain dauer arrest.

### INS-35 modulates dauer arrest cooperatively with INS-7

**Figure 4:**
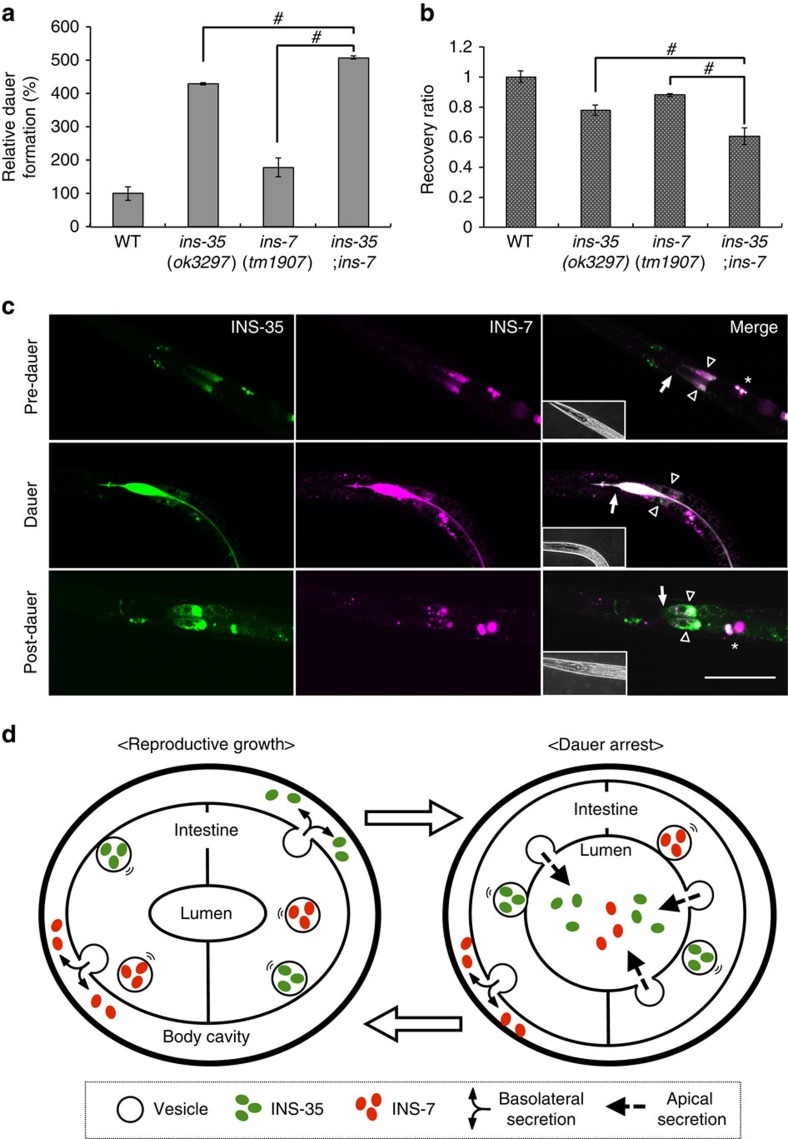
INS-35 modulates dauer arrest cooperatively with INS-7. (**a**,**b**) Relative percentages of dauer formation in the presence of dauer-inducing pheromone (**a**) and ratio of recovery from dauer arrest (**b**) in the mutants, compared with the wild type, are shown. Recovery ratio was measured 12 h after washing out the dauer-inducing pheromone. Data are expressed as the mean±s.e.m. #, *P*<0.05. Error bars are defined as s.e.m. Multiple comparisons between groups were made using Dunnett's test. Detailed parameters including numbers, trials, *P* values, and values of mean±s.e.m. are shown in Supplementary Tables 2a and 3 (**b**). (**c**) Expression patterns of INS-35::VENUS (left), INS-7::mRFP (centre) and merge (right) from pre-dauer to post-dauer stages. Hollow arrowheads indicate anterior intestinal cells. Arrows indicate the intestinal valves. Asterisks indicate coelomocytes. The intestinal canal is located between the intestinal cells (hollow arrowheads). The bright-field images are shown on the bottom left of the merged images. Scale bar, 50 μm. (**d**) Model depicting a secretion switch between reproductive growth (left) and dauer arrest (right).

Finally, to assess whether INS-35 and INS-7 have the same function in the modulation of larval diapause, we determined whether overexpression of one ILP could compensate for the loss of the other ILP. In the presence of dauer-inducing pheromone, the *ins-7* mutant was completely rescued by an *ins-35* transgene (injected at 5 ng μl^−1^). Moreover, the reciprocal experiment showed that the *ins-35* mutant was only partially rescued by an *ins-7* transgene (injected at 15 and 25 ng μl^−1^; Fig. 3c). Therefore, INS-35 and INS-7 are functionally exchangeable in the modulation of larval diapause. These results suggest that both ILPs have the same function in the same tissue. To investigate whether INS-35 modulates larval diapause in cooperation with INS-7, we constructed an *ins-7*; *ins-35* double mutant. In the presence of dauer-inducing pheromone, each *ins-35* or *ins-7* single mutant demonstrated promotion of dauer arrest. Moreover, double mutants showed stronger promotion than that of each single mutant (Fig. 4a). The double mutants also showed suppression of recovery from dauer arrest stronger than each single mutant (Fig. 4b). These results suggest that INS-35 and INS-7 cooperatively modulate larval diapause. We also observed expression patterns of INS-35::VENUS and INS-7::mRFP from pre-dauer to post-dauer stages. As shown in Fig. 4c, INS-35::VENUS and INS-7::mRFP were secreted from intestinal cells into the lumenal space when the worms entered dauer arrest. This altered localization returned to the original localization when the worms returned to developmental growth. These results suggest that larval diapause involves a change in the secretion polarity of INS-35 and INS-7 in the intestine. It has been reported that the intestinal IIS determines dauer versus reproductive development^24^.

### Model for a change of secretory polarity in intestine

Here we propose a model that suggests a role of reversible secretory polarity in the efficient modulation of the IIS pathway for controlling larval diapause in the *C. elegans* intestine (Fig. 4d). To support this model, we investigated (1) the degree to which secretory polarity of ILPs change from basolateral to apical sides, and (2) whether other secretory peptides or proteins show a change of secretory polarity in the intestine between L2–L3 (non-dauer) and dauer stages. It has been reported that DAF-28 secretion into the basolateral side can be quantified as the accumulation of DAF-28::GFP in coelomocytes^25^. Thus, we measured fluorescence intensity of INS-7::mRFP and INS-35::VENUS in coelomocytes to quantify their secretion. At the dauer stage, fluorescence intensity of INS-7::mRFP decreased 32% in coelomocytes. On the other hand, that of INS-35::VENUS decreased 98% there (Supplementary Fig. 8). Therefore, the polarity of INS-7 secretion is partially changed from basolateral to apical sides at the dauer stage. In contrast, the polarity of INS-35 secretion is almost entirely changed from basolateral to apical sides at the dauer stage. Next, to investigate whether other secretory peptides or proteins show a change of secretory polarity in the intestine, we observed the localization of VENUS attached to a secretory signal sequence^19^ (ssVENUS) expressed by the *ges-1* promoter in the intestine. At L2-L3 (non-dauer) stages, ssVENUS expressed in the intestine accumulated in coelomocytes. In contrast, ssVENUS signal was not detected in the coelomocytes at the dauer stage; instead, ssVENUS accumulated in the intestinal lumen (Supplementary Fig. 9). Thus, a change in polarity of secretion is not restricted to ILPs; other secretory peptides or proteins can also change their secretory polarity in the intestine. In mammalian cell cultures, reversible secretory polarity of some peptides has been suggested^26^. Mechanisms for such a change in secretory polarity may be conserved in the animal kingdom. However, the molecules responsible for this reversal in secretory polarity remain unknown. There are many proteins in *C. elegans* that contribute to vesicular transportation and secretion to either the apical membrane or basolateral membrane^27^,^28^. One of them, ASNA-1/ATPase is expressed in the intestine. In addition, knockdown of *asna-1* results in a defect of DAF-28 secretion^29^. Thus, we investigated whether knockdown of *asna-1* affects the intestinal secretion of INS-7 or INS-35 at L2-L3 (non-dauer) and dauer stages. At non-dauer stages, knockdown of *asna-1* resulted in a decrease in fluorescence intensity of INS-7::mRFP and INS-35::VENUS in coelomocytes (Supplementary Fig. 10). At the dauer stage, knockdown of *asna-1* also resulted in a decrease in fluorescence intensity of INS-7::mRFP and INS-35::VENUS in intestinal lumens (Supplementary Fig. 11). These results suggest that ASNA-1 is involved in the secretion of these ILPs to either basolateral side or apical side. The molecular mechanism of switching the secretion polarity between basolateral and apical sides remains elusive. Because of the availability of genetic approaches, the model organism *C. elegans* will be useful for the future analysis of this mechanism and should provide insights into the endocrine regulation of diapause entry and exit.

## Methods

### Strains

*C. elegans* strains were maintained at 20 °C on nematode growth medium (NGM) plates^30^, seeded with *Escherichia coli* OP50 (provided by the Caenorhabditis Genetics Center). *C. elegans* strain N2 is the wild-type strain. Animals containing the following alleles were used in this study: *daf-28*(*tm2308*)V, *ins-6*(*tm2416*)II and *ins-7*(*tm1907*)IV (provided by the National Bioresource Project of Japan for the nematode); *ins-35*(*ok3297*)V (provided by the International *C. elegans* Gene Knockout Consortium); *jcIs1*[*ajm-1*::*gfp*]X and *rrf-3*(*pk1426*)II (provided by the Caenorhabditis Genetics Center). The double mutant animal, *ins-7*(*tm1907*); *ins-35*(*ok3297*), was generated by crossing the respective single mutant animals.

### Dauer formation assay

Dauer formation was tested in the presence of a crude pheromone extract^14^ that induces larval diapause. NGM plates containing 1% crude pheromone extract (v/v) were seeded with *E. coli* OP50 bacteria, and then ∼20 worms were placed on the each plate. *C. elegans* strains were semi-synchronized by allowing gravid adults to lay eggs for 3 h at 20 °C and then picking off the adult worms. After cultivating for 72 h at 25 °C, dauers and non-dauers were identified using a microscope and counted. Dauer larvae were identified by the presence of dark pigment granules, constriction of body and pharynx and loss of pharyngeal pumping. For the dauer formation assays, 3–4 independent plates were assayed as one trial. Multiple comparisons between groups were made using Dunnett's test.

### Dauer exit assay

For preparing dauer larvae, adult worms were placed onto NGM plates containing 10% crude pheromone extract (v/v) seeded with *E. coli* OP50. *C. elegans* strains were semi-synchronized by allowing gravid adults to lay eggs for 3 h at 20 °C and then picking off the adult worms. After cultivating for 72 h at 25 °C, most of the worms had formed dauer larvae, and they were collected from the plates using M9 buffer (85 mM NaCl, 42 mM Na_2_HPO_4_, 22 mM KH_2_PO_4_ and 1 mM MgSO_4_) with 1% SDS. After washing with M9 buffer to remove the pheromone and SDS, the collected worms were placed on fresh NGM plates seeded with *E. coli* OP50 bacteria. After 12 h, dauers and non-dauers were identified and counted. For the dauer exit assays, three independent plates were assayed as one trial. Multiple comparisons between groups were made using Dunnett's test.

### Plasmid Construction

The plasmids described below were constructed as indicated, and used to transform *E. coli* strain DH5α (TOYOBO, Japan), which was used to amplify the plasmids. All primers used in this study are listed in Supplementary Table 4.

*ins-35 transgene (ins-35p::ins-35)*. A genomic fragment containing the *ins-35* coding region and 3.9 kb of the upstream sequence was amplified by PCR from *C. elegans* genomic DNA and inserted into the pBluescript SK (+) vector (Stratagene, USA).

*ins-35 reporter gene (ins-35p::ins-35::venus)*. A genomic fragment containing the *ins-35* coding region and 3.9 kb of the upstream sequence was amplified by PCR from *C. elegans* genomic DNA, and inserted into the pPD_venus vector (kindly provided by Dr Takeshi Ishihara, Kyushu University, Japan), which contains a multiple cloning site followed by *venus* coding sequence and *unc-54* 3′-untranslated region.

*ins-35 promoter::venus*. A 3.9 kb promoter region upstream to a site before the initiator codon was amplified by PCR from *C. elegans* genomic DNA, and inserted into the pPD_venus vector.

*ges-1 promoter::mrfp*. A 3.1 kb promoter region upstream to a site before the initiator codon was amplified by PCR from *C. elegans* genomic DNA and inserted into the pHK_mrfp vector (kindly provided by Dr Hiroshi Kagoshima, National Institute of Genetics, Japan), which contains a multiple cloning site followed by the *mrfp* coding sequence and *unc-54* 3′-untranslated region.

*Tissue-specific transgenes (ins-35 genome)*. A genomic fragment containing the *ins-35* coding region and 0.7 kb of downstream sequence was amplified by PCR from *C. elegans* genomic DNA. In addition, each promoter region of *ges-1* (3.2 kb), *osm-6* (2.5 kb) and *myo-3* (2.5 kb), upstream to a site before the respective initiator codon, was amplified by PCR from *C. elegans* genomic DNA. Each promoter fragment was inserted into the pBluescript SK (+) vector containing the *ins-35* fragment using an In-Fusion HD Cloning Kit (Clontech, USA).

*Tissue-specific transgenes (ins-35 cDNA)*. To construct tissue-specific transgenes (*ins-35* cDNA), the single intron of *ins-35* was removed from the tissue-specific transgenes (*ins-35* genome) using a Q5 site-directed mutagenesis kit (New England BioLabs, USA).

*Tissue-specific reporter genes*. Each fragment (*ges-1p*::*ins-35*, *osm-6p*::*ins-35* and *myo-3p*::*ins-35*), upstream to a site before the stop codon, was amplified by PCR from each tissue-specific transgene and inserted into the pPD_venus vector.

*ges-1p::ins-35 cDNA::venus*. To construct *ges-1p::ins-35* cDNA::*venus*, the single intron of *ins-35* was removed from the *ges-1p*::*ins-35* genome::*venus* plasmid using a Q5 site-directed mutagenesis kit.

*daf-28 reporter gene (daf-28p::daf-28::venus)*. A genomic fragment containing the *daf-28* coding region and 3.5 kb of the upstream sequence was amplified by PCR from *C. elegans* genomic DNA and inserted into the pPD_venus vector.

*ins-7 reporter gene (ins-7p::ins-7::mrfp)*. A genomic fragment containing *ins-7* coding region and 0.6 kb of the upstream sequence was amplified by PCR from *C. elegans* genomic DNA and inserted into the pHK_mrfp vector.

*ins-7 transgene (ins-7p::ins-7)*. A genomic fragment containing *ins-7* coding region and 0.6 kb of the upstream sequence was amplified by PCR and inserted into the pBluescript SK (+) vector.

*ges-1promoter::venus and ges-1promoter::ss::venus*. A 3.1 kb promoter region upstream to a site before the initiator codon was amplified by PCR from *C. elegans* genomic DNA and inserted into the pPD_venus vector. To construct *ges-1p*::*ss*::*venus* plasmid, a 237 bp (first 79aa including signal sequence) of *sel-1* was inserted in between *ges-1* promoter and *venus* coding regions of the *ges-1p*::*venus* plasmid. All plasmids were verified by sequencing.

### Transgenic worms

For rescue (cDNA) experiments, the tissue-specific transgenes (*ins-35* cDNA) were injected at 15 ng μl^−1^ into the gonads^31^ of *ins-35*(*ok3297*), along with pTG96 (ref. 32) (SUR-5::NLS::GFP) as a transformation marker (10 ng μl^−1^). For overexpression experiments, the tissue-specific transgenes (*ins-35* genome) were injected at 15 ng μl^−1^ into the gonads of wild-type animals along with *eft-3p*::*venus* as a transformation marker (10 ng μl^−1^). The transformation markers, *eft-3p*::*venus* and pTF96 did not have any influence on larval diapause. For generating *ins-35p*::*ins-35*::*venus*, *ges-1p*::*ins-35*::*venus*, *osm-6p*::*ins-35*::*venus* or *myo-3p*::*ins-35*::*venus* transgenic worms, each reporter gene was injected at 15 ng μl^−1^ into the gonads of wild-type animals, along with pRF4 [*rol-6*(*su1006*)] (ref. 33) as a transformation marker (20 ng μl^−1^). For generating *daf-28p*::*daf-28*::*venus*, *ins-7p*::*ins-7*::*mrfp*, *ges-1p*::*ins-35* cDNA::*venus*, *ges-1p*:: *venus*, *ges-1p*::*ss*::*venus* or *IDE promoter::venus* transgenic worms, each reporter gene was injected at 25 ng μl^−1^ into the gonads of wild-type animals along with pRF4 [*rol-6*(*su1006*)] (20 ng μl^−1^). For bypass experiments, *ins-7* transgene was injected at 5, 15 and 25 ng μl^−1^ into the gonads of *ins-35*(*ok3297*) animals along with *eft-3p*::*venus* (10 ng μl^−1^). The *ins-35* transgene was injected at 5, 15 and 25 ng μl^−1^ into the gonads of *ins-7*(*tm1907*) animals along with *eft-3p*::*venus* (10 ng μl^−1^).

### Microscopy

Fluorescence images and intensity profiles were obtained with an FV10i confocal laser scanning microscope (Olympus, Japan) and FV10-AWS software (Olympus) for Figs 2, 3, 4 and Supplementary Fig. 5. Fluorescence and differential interference contrast (DIC) images were obtained with an IX71 DIC microscope and DP20 microscope camera (Olympus) for Supplementary Fig. 4. Fluorescence images and intensity profiles were obtained with a Zeiss confocal system (LSM510) equipped with an Axiovert 100M microscope (Carl Zeiss, Germany) for Supplementary Figs 7, 8, 10 and 11. Fluorescence and DIC images were obtained with Axioskop microscope and AxioCam MR microscope camera (Carl Zeiss) for Fig. 1 and Supplementary Figs 3, 7, 8, 9, 10 and 11.

### An anti-INS-35 antibody and Western blotting

A rabbit anti-INS-35 polyclonal antibody was generated by immunizing animals with synthetic peptide (Ac-HHKMDENAFGINNRHC) conjugated to Keyhole limpet haemocyanin (KLH) (GL Biochem Ltd, China). The synthetic peptide corresponds to His6 to Cys21 in the putative mature INS-35 (ref. 6). For preparing dauer larvae, we prepared NGM plates containing 10% (v/v) crude pheromone extract seeded with *E. coli* OP50 bacteria, and then gravid worms were placed onto 2–3 of these plates, ∼20 worms per plate. For semi-synchronization, the worms were cultured for 3 h at 20 °C to lay eggs and then picking off the gravid worms. After cultivating for 72 h at 25 °C, most of the worms formed dauer larvae, and they were collected from the plates using M9 buffer with 1% SDS. After washing with M9 buffer, dauer larvae were transferred onto fresh NGM plates containing 10% (v/v) crude pheromone extract without bacteria to maintain diapause, and then cultured for 1, 7 or 14 days at 25 °C. We prepared worm lysates from approximately 200 dauer larvae in a volume of 20 μl using the protocol of Hannak *et al*^34^.

A portion of the sample (10 μl) was loaded onto a Mini-PROTEIN TGX 10% SDS–polyacrylamide gel (Fig. 2c) or a Mini-PROTEIN TGX Any kD SDS–polyacrylamide gel (Supplementary Fig. 6c–e) (Bio-Rad Laboratories, Inc, USA). After running the gel, the separated proteins were transferred to a nitrocellulose membrane (Bio-Rad Laboratories, Inc) using transfer buffer (25 mM Tris-Base, 192 mM glycine and 20% methanol (v/v)) at 100 V for 1 h. Monoclonal anti-actin antibody (C4; Abnova, Taiwan), polyclonal anti-INS-35 antibody or monoclonal anti-GFP antibody (mFX75; Wako, Japan) were diluted 1:1,000 in TBST buffer (5% skim-milk (w/v), 0.1% Tween-20 (v/v), 1 × TBS (pH 7.6)), and incubated with the membrane for 1 h at room temperature. The primary antibodies were detected using anti-mouse horse radish peroxidase-conjugated secondary antibody (CAT#: NXA931), or anti-rabbit horse radish peroxidase-conjugated secondary antibody (CAT#: NA9340) (GE Healthcare UK Ltd, England). These secondary antibodies were diluted 1:5,000. The signal from the membrane was obtained using a Pierce ECL Western Blotting Substrate (Thermo SCIENTIFIC) and HyBlot CL film (DENVILLE SCIENTIFIC Inc USA).

### RNAi experiments

RNAi-mediated gene knockdown experiments were performed as described by Kamath *et al.*^35^ Briefly, RNAi was performed by the feeding method. Gravid worms were placed on

NGM plates containing 200 μM isopropyl-β-D-thiogalactopyranoside (IPTG), 20 μg ml^−1^ ampicillin and 1% crude pheromone extract (v/v) seeded with the specified worm strain bacteria expressing double-strand RNA. After 3 h at 20 °C, the worms were picked off. Remaining eggs were cultured for 72 h at 25 °C until the animals reached dauer or non-dauer (L2–L3) stages.

Supplementary Table 1: Each cDNA fragment from type-α insulin-like genes was amplified from *C. elegans* cDNA by PCR and cloned into the RNAi vector pPD129.36 (kindly provided by Dr Andrew Fire, Stanford University, USA). The RNAi plasmids and empty vector (as a control) were transformed into *E. coli* strain HT115(DE3) (provided by the Caenorhabditis Genetics Center), and then each resulting strain was seeded onto NGM plates containing 200 μM IPTG, 20 μg ml^−1^ ampicillin and 1% crude pheromone extract (v/v).

Supplementary Figs 10 and 11: A fragment of *asna-1* cDNA was amplified from *C. elegans* cDNA by PCR and cloned into the RNAi vector pPD129.36. The RNAi plasmid and empty vector were transformed into *E. coli* strain HT115(DE3), and then each resulting strain was seeded onto NGM plates containing 200 μM IPTG and 20 μg ml^−1^ ampicillin. For Supplementary Fig. 11, we used the NGM plates containing 10% crude pheromone extract (v/v) to induce larval diapause.

## Additional information

**How to cite this article**: Matsunaga, Y. *et al.* Diapause is associated with a change in the polarity of secretion of insulin-like peptides. *Nat. Commun.* 7:10573 doi: 10.1038/ncomms10573 (2016).

## Supplementary Material

Supplementary Information Supplementary Figures 1-11 and Supplementary Tables 1-4
