# Supplementary material for: Diapause is associated with a change in the polarity of secretion of insulin-like peptides
Source: Nat Commun. 2016 Feb 3;7:10573. doi: 10.1038/ncomms10573 (PMC4742890; doi:10.1038/ncomms10573)
Supplement: Supplementary Information — Supplementary Figures 1-11 and Supplementary Tables 1-4 [file ncomms10573-s1.pdf]

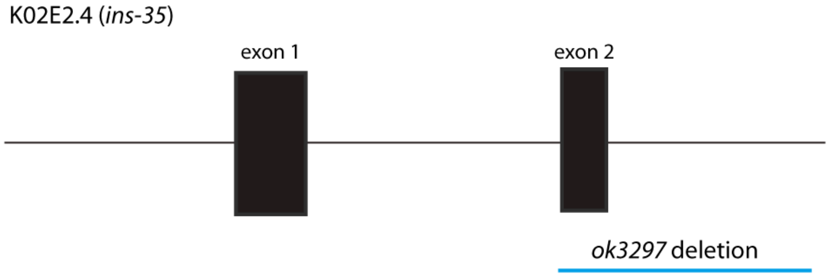


```
------atttaagtccaaattttcca------------ aaattggaaatttccggcaa --- wild-type
------atttaagtccaaattttcca [664 bp deletion] aaattggaaatttccggcaa --- ok3297
```

Supplementary Figure 1 | Location of deletion in *ins-35*(*ok3297*). Black boxes represent exons in *ins-35*. The blue line indicates the *ins-35*(*ok3297*) deletion site. Sequencing revealed the indicated break point for *ins-35*(*ok3297*).

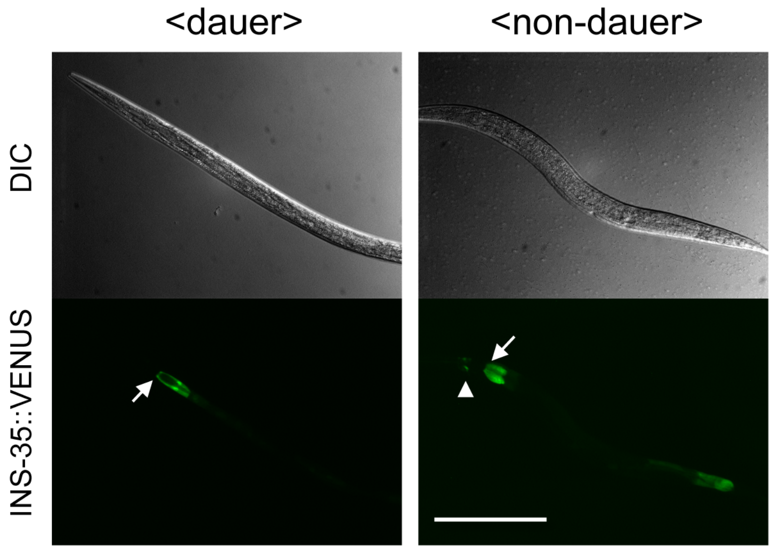

Supplementary Figure 2 | Expression patterns of transgenic worms expressing *ins-35p*::VENUS. Localization of *ins-35p*::VENUS at L2-L3 (non-dauer) (right) and the day 1 dauer (left) stages. Arrows indicate the intestinal valves. Arrowhead indicates head neuron. The scale bar is 100 μm.

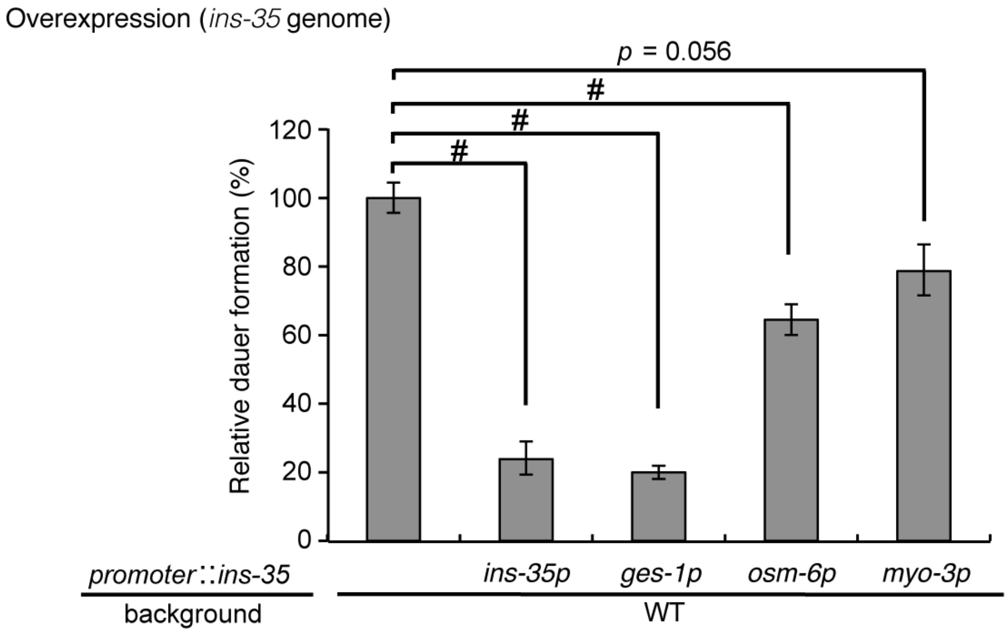

Supplementary Figure 3 | Relative percentages of dauer formation in each *ins-35* mutant in the presence of dauer inducing-pheromone, compared with wild-type animals (overexpression experiment). Data are expressed as the mean ± S.E. $^{\#}p <$ 0.05; N.S.: Not Significant. Error bars are defined as s.e.m.. Multiple comparisons between groups were made using Dunnett's test. Detailed parameters including numbers, trials, *p*-values, and values of mean ± S.E. are shown in Supplementary Table 2.

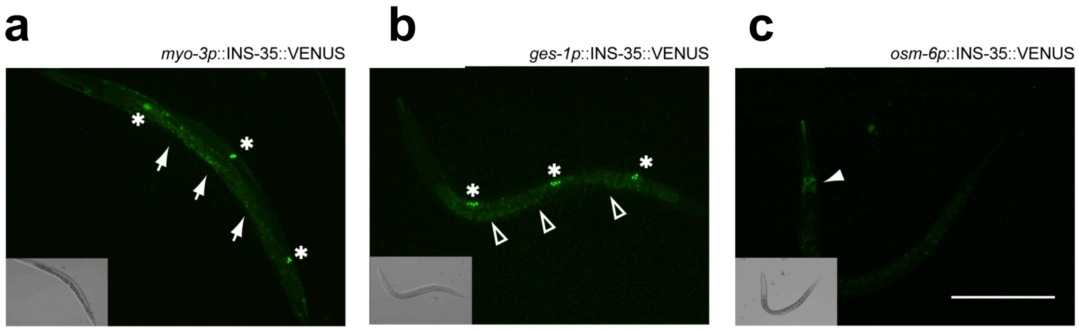

Supplementary Figure 4 | Expression patterns of INS-35::VENUS driven by tissue-specific promoters at the L2 stage. Expression patterns of *myo-3p*::INS-35::VENUS (a), *ges-1p*::INS-35::VENUS (b) and *osm-6p*::INS-35::VENUS (c) are shown. Arrows indicate muscle cells. Hollow arrowheads indicate intestinal cells. The solid arrowhead indicates amphid neurons. Asterisks indicate coelomocytes. The DIC images are shown on the bottom left. The scale bar is 25 μm.

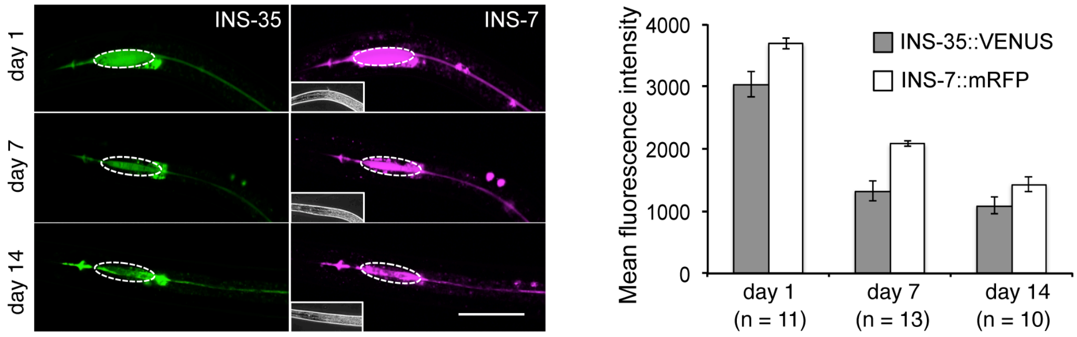

Supplementary Figure 5 | Fluorescence intensities of INS-35::VENUS and INS-7::mRFP at day 1, day 7 and day 14 dauers. The mean fluorescence intensities of INS-35::VENUS driven by the *ins-35* promoter and INS-7::mRFP driven by the *ins-7* promoter were calculated within the dashed ellipsoidal areas. Each box represents the intensity of INS-35::VENUS (gray) and INS-7::mRFP (white). Scale bar, 50 µm. Data are expressed as the mean ± S.E. Error bars are defined as s.e.m..

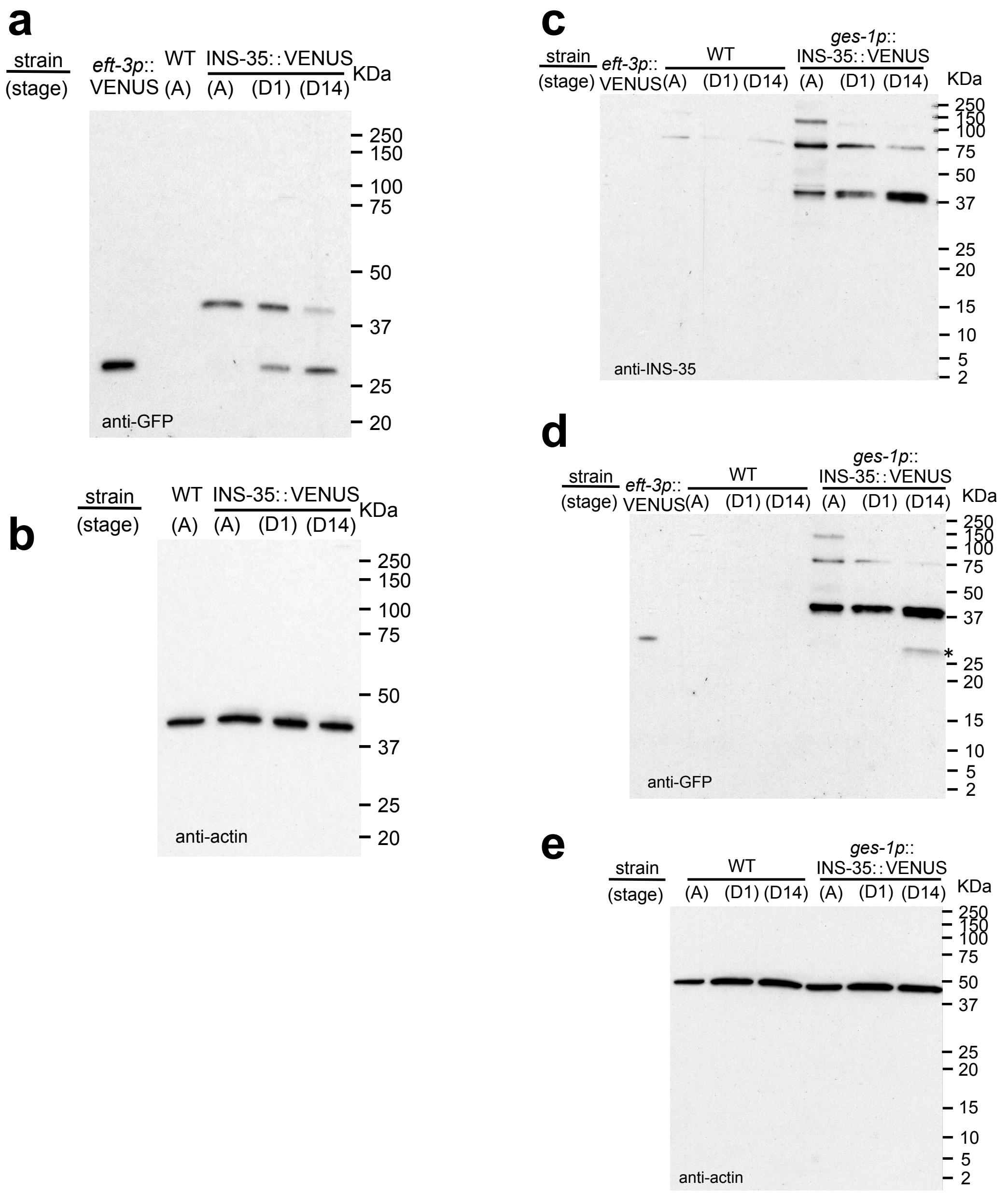

Supplementary Figure 6 | Western blot analysis of extractions of *ins-35p*::*ins-35*::*venus* (a and b) or *ges-1p*::*ins-35*::*venus* (c, d, and e) expressing worms. Asterisk indicates a band of approximately 31 kDa in the lane corresponding to individuals at day 14 of dauer arrest. A, adult stage; D1, dauer stage at day 1; D14, dauer stage at day 14.

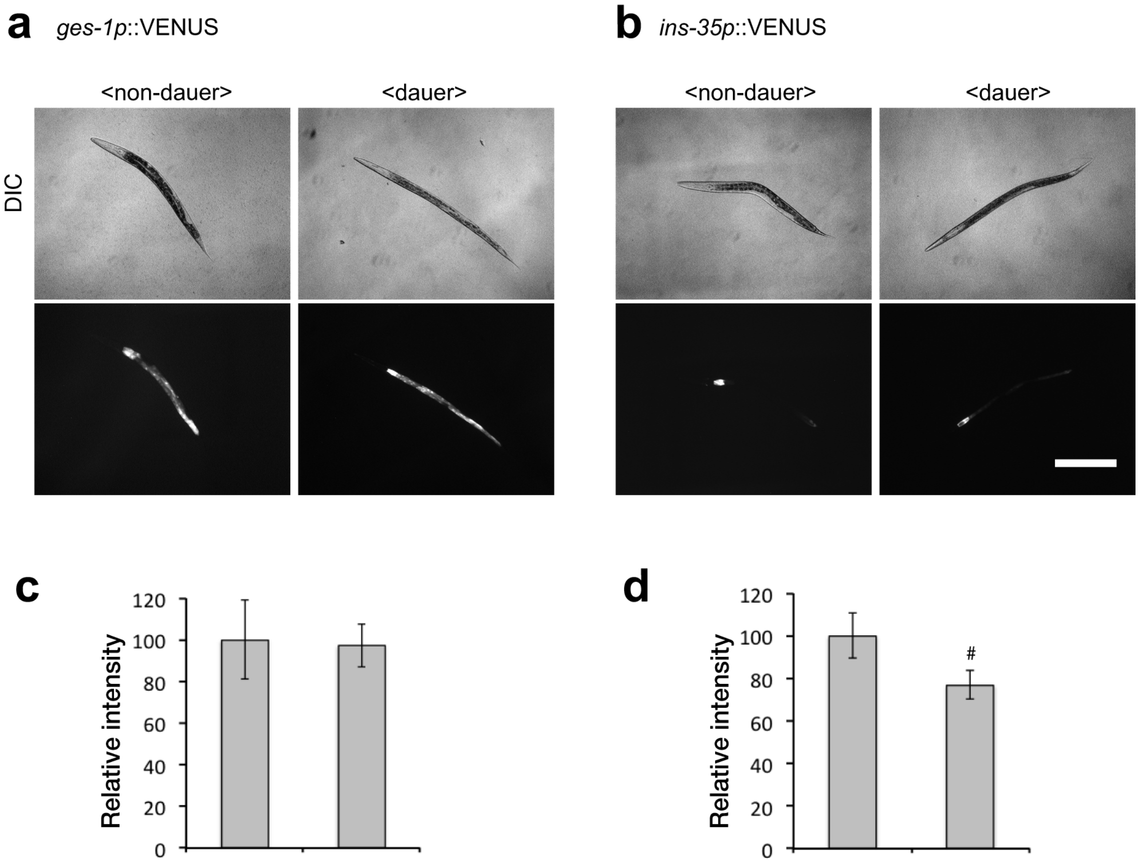

Supplementary Figure 7 | Fluorescent intensity of *ins-35p*::VENUS is decreased in the day 1 dauer stage. (a and b) Panels show DIC and fluorescence images of *ges-1p*::VENUS (a) and *ins-35p*::VENUS (b) expressing worms at non-dauer (L2-3) and day 1 dauer stages. Scale bar is 100 μm. (c and d) Relative intensity of *ges-1p*::VENUS (c) and *ins-35p*::VENUS (d) in the worms. Data are expressed as the mean $\pm$ S.E. # $p < 0.05$. n=10. Error bars are defined as s.e.m.. Trials=1. Comparisons between two groups were made using Student's *t*-test.

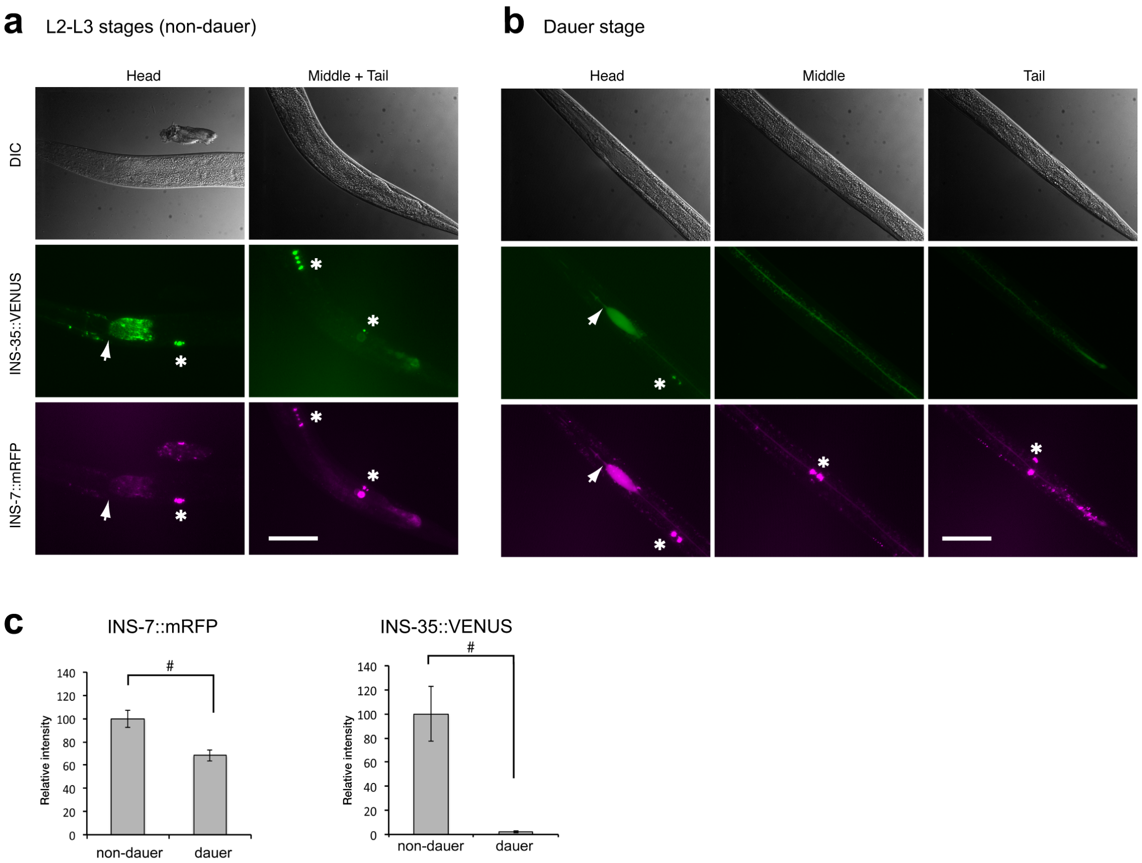

Supplementary Figure 8 | Comparison of fluorescence intensity in coelomocytes between L2-L3 (non-dauer) and the day 1 dauer stages. Localization of INS-35::VENUS driven by the *ins-35* promoter and INS-7::mRFP driven by the *ins-7* promoter at (a) L2-L3 (non-dauer) and (b) the day 1 dauer stages. Arrows indicate the intestinal valves. Asterisks indicate coelomocytes. The scale bar is 50 µm. (c) Relative intensity of INS-7::mRFP and INS-35::VENUS in coelomocytes. Data are expressed as the mean ± S.E. # $p < 0.05$. n=10. Error bars are defined as s.e.m.. Trials=1. Comparisons between two groups were made using Student's *t*-test.

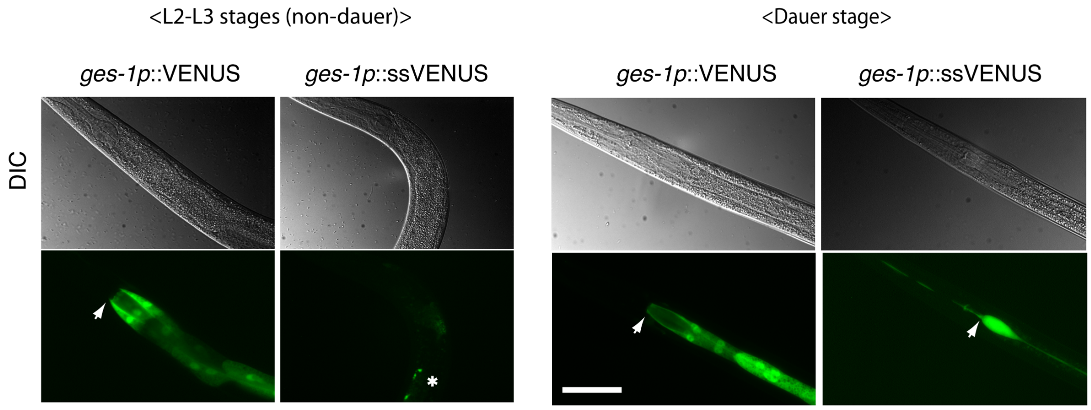

Supplementary Figure 9 | Localization of *ges-1p*::VENUS and *ges-1p*::ssVENUS at L2-L3 (non-dauer) and the day 1 dauer stages. In the *ges-1p*::*venus* expressing worms, VENUS signal in coelomocytes was not detected at non-dauer or the dauer stages. ssVENUS was detected in coelomocytes at L2-L3 (non-dauer) stages and accumulated in the intestinal canal at the day 1 dauer stage. Arrows indicate the intestinal valves. The asterisk indicates a pair of coelomocytes. The scale bar is 50 μm.

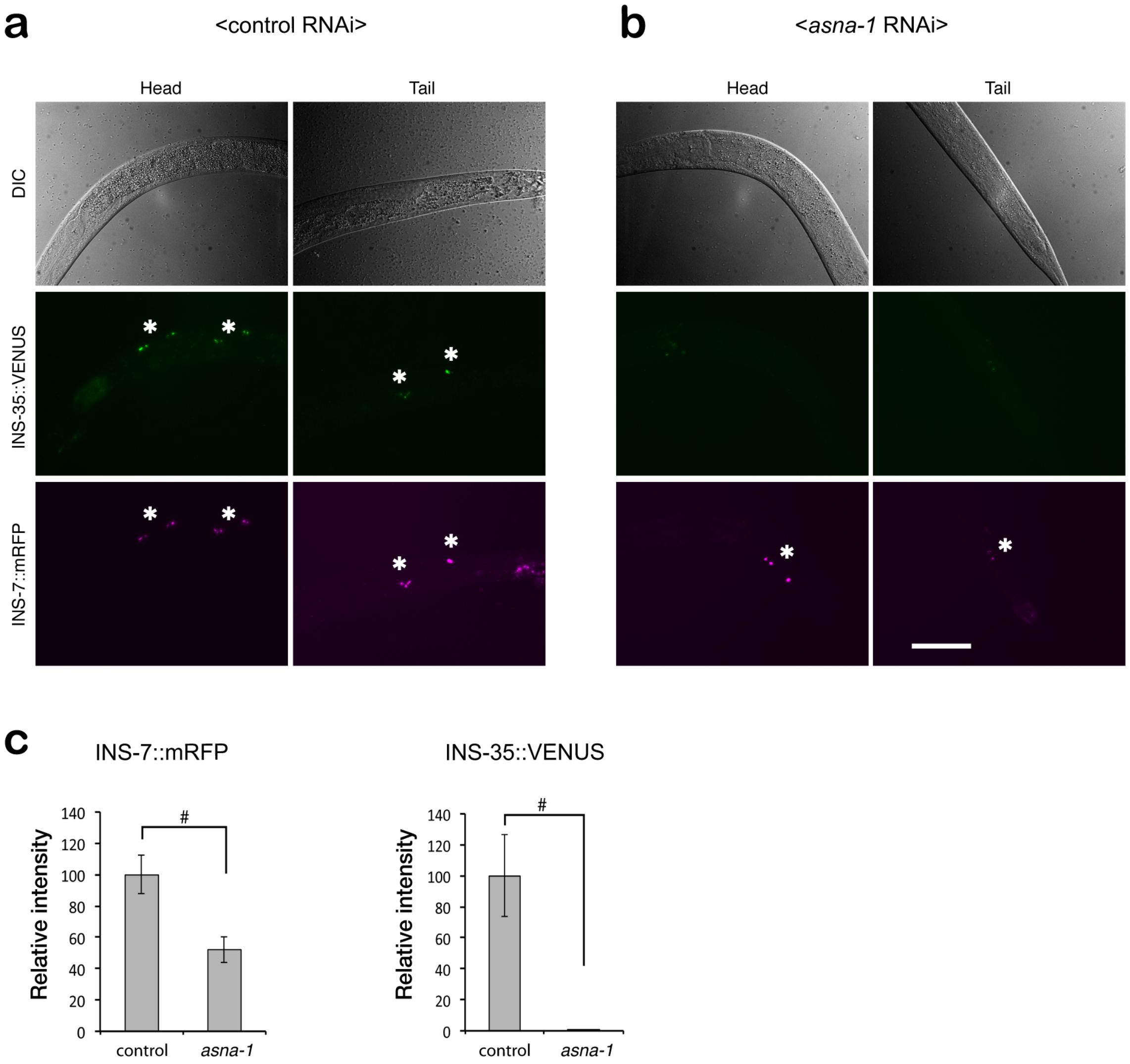

Supplementary Figure 10 | *asna-1* RNAi decreases fluorescent intensities of INS-35::VENUS and INS-7::mRFP in coelomocytes at L2-L3 stages (non-dauer). Localization of INS-35::VENUS driven by the *ins-35* promoter and INS-7::mRFP driven by the *ins-7* promoter under control RNAi (a) and *asna-1* RNAi (b) conditions. Asterisks indicate coelomocytes. The scale bar is 50 µm. (c) Relative intensity of INS-7::mRFP and INS-35::VENUS in coelomocytes. Data are expressed as the mean ± S.E. # $p < 0.05$. n=10. Error bars are defined as s.e.m.. Trials=1. Comparisons between two groups were made using Student's *t*-test.

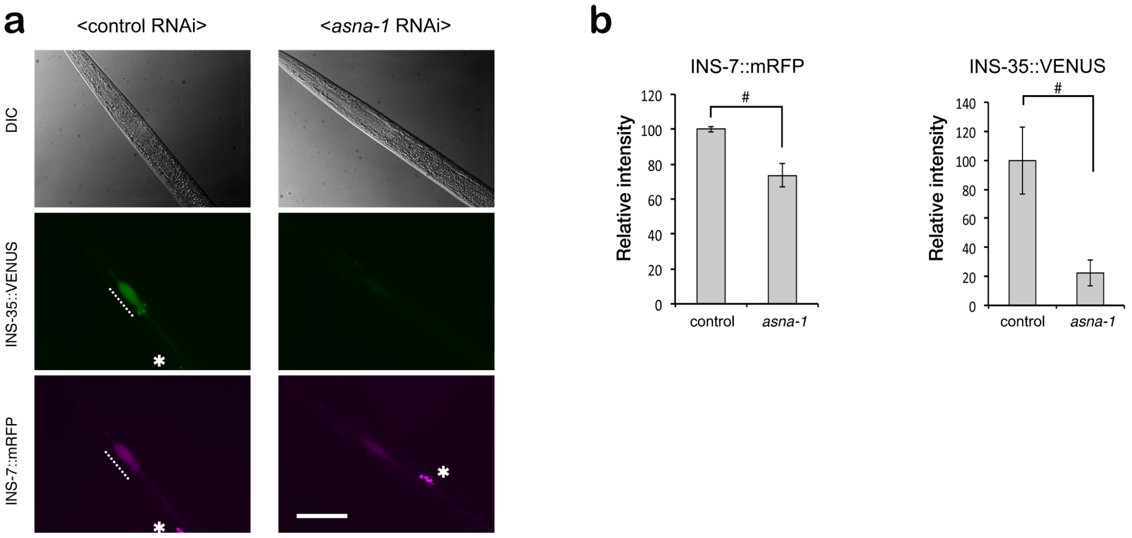

Supplementary Figure 11 | *asna-1* RNAi decreases fluorescent intensities of INS-35::VENUS and INS-7::mRFP in the intestinal lumen at the day 1 dauer stage. (a) Localization of INS-35::VENUS driven by the *ins-35* promoter and INS-7::mRFP driven by the *ins-7* promoter under control RNAi (Left) and *asna-1* RNAi (right). Asterisks indicate coelomocytes. Dashed lines indicate anterior intestinal lumens. The scale bar is 50 μm. (b) Relative intensity of INS-7::mRFP and INS-35::VENUS in the anterior intestinal lumen. Data are expressed as the mean ± S.E. # $p < 0.05$. n=10. Error bars are defined as s.e.m.. Trials=1. Comparisons between two groups were made using Student's *t*-test.

Supplementary Table 1 | Relative dauer formation of *ins*-RNAi treated aniamls

Target gene	Relative dauer formation (%)	Number dauer	Number non-dauer	Number total (trials)	*p* value
control	100.0 ± 10.2	893	813	1706 (7)	control
ins-20	91.7 ± 22.1	250	306	556 (2)	0.999
ins-21	105.1 ± 8.9	378	427	805 (4)	1.000
ins-22	83.8 ± 21.0	315	411	726 (4)	0.976
ins-23	83.6 ± 20.7	354	589	943 (4)	0.677
ins-24	102.0 ± 16.0	250	169	419 (2)	0.999
ins-25	132.2 ± 4.7	101	114	215 (1)	1.000
ins-27	121.6 ± 11.0	401	293	694 (3)	0.999
ins-28	122.6 ± 3.9	268	182	450 (2)	0.996
ins-29	102.4 ± 9.4	286	333	619 (3)	0.999
ins-30	96.6 ± 19.1	148	293	441 (2)	0.606
ins-31	112.2 ± 10.7	270	162	432 (2)	0.998
ins-33	139.0 ± 9.6	388	174	562 (2)	0.450
ins-34	79.1 ± 21.1	402	306	708 (2)	1.000
ins-35	140.4 ± 6.5	502	143	645 (3)	0.022
ins-36	89.5 ± 5.5	115	88	203 (1)	1.000

Relative percentages of dauer formation in each ins-RNAi treated animals compared with control RNAi treated animals are shown. Data are expressed as the mean ± S.E.. For the dauer formation assays, 3-4 independent plates were assayed as 1 trial. Multiple comparisons between groups were made using Dunnett’s test.

Supplementary Table 2 | Summary of the relative dauer formation

Background	Transgene (Conc.)	Relative dauer formation (%)	Number dauer	non-dauer	total (trials)	*p* value
Figure 1c						
Wild type		100.0 ± 3.7	85	234	319 (2)	control
ins-35(*ok3297*)		327.1 ± 3.7	285	41	326 (2)	<.001
ins-35(*ok3297*)	*ins-35p*::*ins-35* **cDNA** (15 ng/μl)	64.1 ± 6.9	27	130	157 (2)	0.031
ins-35(*ok3297*)	*ges-1p*::*ins-35* **cDNA** (15 ng/μl)	9.0 ± 2.5	7	180	187 (2)	<.001
ins-35(*ok3297*)	*osm-6p*::*ins-35* **cDNA** (15 ng/μl)	127.6 ± 5.7	60	116	176 (2)	0.135
ins-35(*ok3297*)	*myo-3p*::*ins-35* **cDNA** (15 ng/μl)	55.1 ± 9.5	56	139	195 (2)	0.005
Supplementary Figure 2						
Wild type		100.0 ± 4.5	105	169	274 (2)	control
Wild type	*ins-35p*::*ins-35* (15 ng/μl)	24.1 ± 4.8	32	308	341 (2)	<.001
Wild type	*ges-1p*::*ins-35* (15 ng/μl)	20.0 ± 1.8	23	263	286 (2)	<.001
Wild type	*osm-6p*::*ins-35* (15 ng/μl)	64.5 ± 4.4	81	246	327 (2)	<.001
Wild type	*myo-3p*::*ins-35* (15 ng/μl)	78.9 ± 7.6	96	227	323 (2)	0.056
Figure 3a						
Wild type		100.0 ± 7.3	42	263	305 (3)	control
ins-6(*tm2416*)		95.1 ± 6.2	35	236	271 (3)	0.994
ins-7(*tm1907*)		243.2 ± 15.3	104	211	315 (3)	<.001
ins-35(*ok3297*)		645.7 ± 15.7	209	30	239 (3)	<.001
daf-28(*tm2308*)		620.1 ± 8.6	323	61	384 (3)	<.001
Figure 3c						
Wild type		100.0 ± 5.0	172	263	435 (2)	<.001
ins-7(*tm1907*)		166.5 ± 4.4	402	216	618 (2)	control
ins-7(*tm1907*)	*ins-35p*::*ins-35* (25 ng/μl)	11.5 ± 3.1	17	298	315 (2)	<.001
ins-7(*tm1907*)	*ins-35p*::*ins-35* (15 ng/μl)	29.2 ± 3.2	46	349	395 (2)	<.001
ins-7(*tm1907*)	*ins-35p*::*ins-35* (5 ng/μl)	77.4 ± 4.7	106	230	336 (2)	<.001
Wild type		100.0 ± 8.2	87	420	507 (2)	<.001
ins-35(*ok3297*)		447.1 ± 7.6	377	123	500 (2)	control
ins-35(*ok3297*)	*ins-7p*::*ins-7* (25 ng/μl)	200.8 ± 8.8	137	271	408 (2)	0.022
ins-35(*ok3297*)	*ins-7p*::*ins-7* (15 ng/μl)	288.4 ± 8.4	212	222	434 (2)	<.001
ins-35(*ok3297*)	*ins-7p*::*ins-7* (5 ng/μl)	404.3 ± 12.8	265	126	391 (2)	<.001
Figure 4a						
Wild type		100.0 ± 20.2	55	238	293 (3)	<.001
ins-35(*ok3297*)		428.8 ± 4.0	169	41	210 (3)	0.034
ins-7(*tm1907*)		178.4 ± 27.8	82	164	246 (3)	<.001
ins-7; *ins-35*		493.8 ± 13.7	229	13	242 (3)	control

Data are expressed as the mean ± S.E.. For the dauer formation assays, 3-4 independent plates were assayed as 1 trial. Multiple comparisons between groups were made using Dunnett's test.

Supplementary Table 3 | Summary of the recovery ratio

Background	Recovery ratio (%)	Number dauer	non-dauer	total (trials)	*p* value
Wild type	100.0 ± 3.9	104	460	564 (2)	<.001
ins-35(*ok3297*)	77.6 ± 3.3	138	393	531 (2)	0.029
ins-7(*tm1907*)	87.7 ± 1.0	203	269	472 (2)	0.002
ins-7; *ins-35*	60.3 ± 5.4	236	233	469 (2)	control

Data are expressed as the mean ± S.E.. For the dauer exit assays, 3 independent plates were assayed as 1 trial. Multiple comparisons between groups were made using Dunnett's test.

Supplementary Table 4 | PCR primers used in this study

primer name		sequence 5' to 3'	
		Fw	Rv
ins-35(ok3297)		gattctcatggaaggatgtg	aagttcactgagcattcagg
ins-35p::*ins-35*		ataggtaccagttcacggttatcaatgag	taagtcgactcaatataaatatctgcagg
ins-35p::*ins-35*::*venus* or *mrfp*		atagcatgctttcacgtacctctaaggaag	tatggatccaattgaatagttcatctgagtc
ins-35p::*venus*		atagcatgctttcacgtacctctaaggaag	tatggatcctgctggaaaattagacaatttc
ges-1p::*mrfp* or *venus*		attgcatgcagaccatacggaaatagctgttag	aatcccgggctgaattcaaagataagatatgtaatag
osm-6p::*ins-35*	*osm-6 region*	atcgataagcttgattaacgggctgatcgaacag	gaatatttgcttcatagatgtatactaatgaagg
	ins-35 region	atgaagcaaatattcttggtaatccttgctg	ctgcaggaattcgatcaatataaatatctgcagg
ges-1p::*ins-35*	*ges-1 region*	atcgataagcttgattcaccaatacctttagtgacgat	ctgaattcaaagataagatatgtaatagatttttgaagc
	ins-35 region	tatctttgaattcagatgaagcaaatattcttggtaat	ctgcaggaattcgatcaatataaatatctgcagg
myo-3p::*ins-35*	*myo-3 region*	atcgataagcttgatcggctataataagttcttgaat	gaatatttgcttcattctagatggatctagtggtcg
	ins-35 region	atgaagcaaatattcttggtaatccttgctg	ctgcaggaattcgatcaatataaatatctgcagg
deletion of intron in *ins-35*		ATTCTCATGGAAGGATGTGGCTC	CTTTTCATAGTTCTGACATATTGCTC
daf-28p::*daf-28*::*venus*		actgcatgcgatgcttatcaatagcggatatacg	tatggatccacaagaagcaaacgtgggcaac
ins-7p::*ins-7*::*mrfp*		taagcttcttcgaaggataaccccgaag	tggatccaaggacagcactgttttcg
ins-7p::*ins-7*		attGTCGACagttacaccaatgcctattc	ttaCTGCAGacatagcgcgagatcagtttg
sel-1		accgatatcATGATTAAAACCTATCTGACACTG	accgatatcAGGGCTTGGTGGTTGTTCTA
C02G6.1p::*venus*		gttGCATGCttgagtgcagacagtacgac	ctaCCCGGGcatttttcaccaaaaaatagtgctc
C02G6.2p::*venus*		gttGCATGCgttctgccgcagaagcagtg	ctaCCCGGGcattttagcagaaaactagactcggtg
C28F5.4p::*venus*		gttGCATGCgagataccgacatgcttctg	ctaCCCGGGcattcaggataaaactcgacaatc
F44E7.4p::*venus*		caagcatgctgaaaacattgccacacttc	ctaCCCGGGcattcgattgctctgtaaatttc
Y70C5C.1p::*venus*		gttGCATGCatattttccacgccatttgttc	ctaCCCGGGcatttttagaagagaggttaaatctttg
ins-20 RNAi		tggacaaaccatcctacctg	tcattctggacagcaccgtt
ins-21 RNAi		gaaaacctactcatttttcgtg	cgggacagcaaatgtacttg
ins-22 RNAi		atgcacactacaactattctc	tcattggttatcacagcacat
ins-23 RNAi		gttcgttcttcttattattctc	tcactgagaagttggacag
ins-24 RNAi		gagatctcccaccttgtttc	tagaaaacgaagccagatgg
ins-25 RNAi		gggcaacacatattcttcag	cagctttctgaagccaaacc
ins-26 RNAi		agagctctcgtcgctattct	gtgaatgaatagcttcatggt
ins-27 RNAi		cttccgcttaatcttgctct	gatgagaaagttgggtcttc
ins-28 RNAi		atgatgcgctcattctttgt	agattggacagcacatttca
ins-29 RNAi		gttctgtaaatttgtattggtg	caagcaagatttgaaggacag
ins-30 RNAi		tgagttctcacgccctggt	cagaagacgaatggtgtctg
ins-31 RNAi		gaagatgcccttgatcttgc	aggcttatgctgtgtgcaga
ins-32 RNAi		tcgattctgttgatccttcta	atcagggcacattattgatttg
ins-33 RNAi		cctgcttaatcctgcttctg	atcctcctttggacagcaac
ins-34 RNAi		tgcaaattcggtccatcgtg	ttgctcattggttggacgac
ins-35 RNAi		gaagcaaatattcttggtaatc	gaatagttcatctgagtcaac
ins-36 RNAi		atgaacataggcaaatgttcc	ttgaacctgcatttgggcag
asna-1 RNAi		atgtcggatcagctggaagcctcta	tagcttcaggatccaccatccgttc
